# Single-crystalline δ-Ni_2_Si nanowires with excellent physical properties

**DOI:** 10.1186/1556-276X-8-290

**Published:** 2013-06-19

**Authors:** Wen-Li Chiu, Chung-Hua Chiu, Jui-Yuan Chen, Chun-Wei Huang, Yu-Ting Huang, Kuo-Chang Lu, Cheng-Lun Hsin, Ping-Hung Yeh, Wen-Wei Wu

**Affiliations:** 1 Department of Materials Science and Engineering, National Chiao Tung University, Hsinchu, 300, Taiwan; 2 Department of Materials Science and Engineering, National Cheng Kung University, Tainan, 701, Taiwan; 3 Department of Electrical Engineering, National Central University, Tao Yuan, 320, Taiwan; 4 Department of Physics, Tamkang University, New Taipei City, 25137, Taiwan

**Keywords:** CVD, Ni_2_Si nanowires, Field emission, Ferromagnetic characteristic

## Abstract

In this article, we report the synthesis of single-crystalline nickel silicide nanowires (NWs) via chemical vapor deposition method using NiCl_2_·6H_2_O as a single-source precursor. Various morphologies of δ-Ni_2_Si NWs were successfully acquired by controlling the growth conditions. The growth mechanism of the δ-Ni_2_Si NWs was thoroughly discussed and identified with microscopy studies. Field emission measurements show a low turn-on field (4.12 V/μm), and magnetic property measurements show a classic ferromagnetic characteristic, which demonstrates promising potential applications for field emitters, magnetic storage, and biological cell separation.

## Background

With the miniaturization of electronic devices, one-dimensional (1-D) nanostructures have attracted much attention due to their distinct physical properties compared with thin film and bulk materials. One-dimensional materials, such as nanorods, nanotubes, nanowires (NWs), and nanobelts, are promising to be utilized in spintronics, thermoelectric and electronic devices, etc. [1-5]. Metal silicides have been widely synthesized and utilized in the contemporary metal-oxide-semiconductor field-effect transistor as source/drain contact materials, interconnection [6], and Schottky barrier contacts. One-dimensional metal silicides have shown excellent field emission [7,8] and magnetic properties [9-11]. Hence, recently, the synthesis and study of 1-D metal silicide nanostructures and silicide/silicon or silicide/siliconoxide nanoheterostructures have been extensively investigated [9,12-18]. Among various silicides, Ni silicide NWs with low resistivity, low contact resistance, and excellent field emission properties [19,20] are considered as a promising material in the critical utilization for the future nanotechnology. Thus, plenty of methods have been reported to synthesize Ni silicide NWs. Wu et al. have formed NiSi NWs by the chemical reaction between coated Ni metal layers and pre-fabricated Si NWs [13]. In addition, metal-induced growth, chemical vapor deposition (CVD), and chemical vapor transport method have been successfully applied to synthesize NiSi [21,22], Ni_31_Si_12_[20], Ni_3_Si [23], and Ni_2_Si [24] NWs, and their physical properties have been investigated. For simplification of the whole processing, metal chloride compounds such as Fe(SiCl_3_)_2_(CO)_4_[9], CoCl_2_[11,25], or NiCl_2_[19] are commonly used as single-source precursors (SSPs) in synthesizing metal-silicide NWs. In this work, δ-Ni_2_Si NWs were synthesized via CVD method with SSP of NiCl_2_. The morphology and yield of δ-Ni_2_Si NWs can be mastered through parameter control. The δ-Ni_2_Si NWs were structurally characterized via high-resolution transmission electronic microscopy (HRTEM). The growth mechanisms of δ-Ni_2_Si NWs and NiSi phases were identified through structural analysis by X-ray diffraction (XRD) and TEM. Electrical measurements showed an outstanding field emission property, and magnetic property measurements demonstrated a classic ferromagnetic behavior of the δ-Ni_2_Si NWs.

## Methods

The synthesis of the silicide NWs was carried out in the three-zone furnace via a chemical vapor deposition process. Commercial single-crystalline Si substrates were firstly cleaned in acetone for 10 min by ultrasonication. In order to remove the native oxide layer, substrates were dipped in dilute HF solutions for 30 s and then dried by nitrogen gas flow. The nickel chloride (NiCl_2_) precursor was placed in an aluminum boat at the upstream and flown by carrier gas Ar at 30 sccm, while Si substrates were put at the downstream. The temperatures of the precursor and substrates were controlled at 600°C and 400°C, respectively, and held for 15 to 30 min with a 10°C/min ramping rate. The vacuum pressure was controlled in the range of 6 to 15 Torr. The morphologies were investigated by field emission scanning electron microscopy. XRD and TEM were utilized in structural characterization. The noise of the atomic images was filtered by fast Fourier transform (FFT). The field emission property was measured using a Keithley power supply (Keithly Instruments Inc., Cleveland, OH, USA) with an anode probe of 180 μm in diameter. A superconductive quantum interference device (SQUID; MPMS XL, SQUID Technology, Heddington, Wiltshire, UK) was utilized for magnetic property measurements.

## Results and discussion

**Figure 1:**
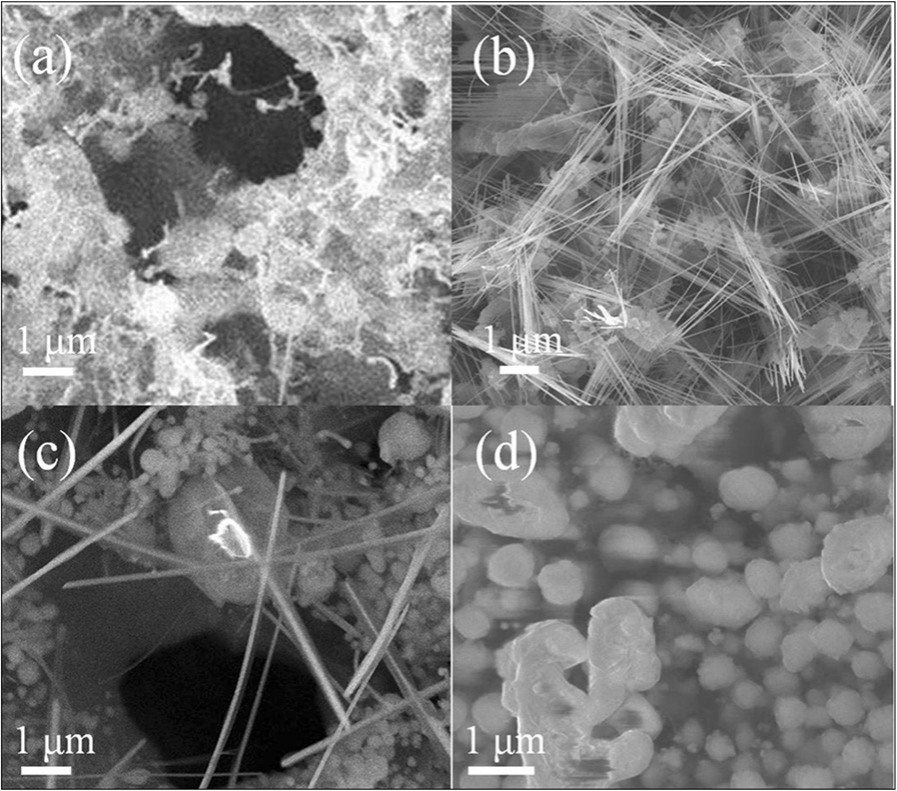
**SEM images of as-synthesized NWs at vacuum pressures of (a) 6, (b) 9, (c) 12, and (d) 15 Torr.** The temperature was fixed at 400°C, reaction time was 30 min, and carrier gas flow rate was held at 30 sccm.

Figure 1a,b,c,d shows the SEM images of samples grown at different pressures (6, 9, 12, 15 Torr, respectively), indicating that the geometry on the surface of substrates varied with the ambient condition. With lower partial pressure of the precursor, as shown in Figure 1a, Ni silicide NWs were not formed due to insufficient supply of the Ni source; however, small nanowhiskers can be observed on the surface. As the ambient pressure was raised to the range of 9 to 12 Torr (Figure 1b,c), NWs with high aspect ratios were obtained for proper concentrations of precursors and growth conditions. The diameter of the NWs slightly increased with the increase of the ambient pressure (from 30 to 50 nm to 40 to 70 nm). This may be attributed to the fact that higher precursor concentration is more suitable for the formation of δ-Ni_2_Si system. Furthermore, when the pressure was higher than 15 Torr, the concentration of the Ni source was oversaturated and the morphology of the product turned into islands instead of NWs. Those islands may result from the condition change to decrease the surface energy of the system by transforming into bulk-like structures, as shown in Figure 1d. Thus, the diameter of the NWs can be controlled under specific pressure range and the ambient pressure plays an important role in maintaining the morphology of the NWs.

**Figure 2:**
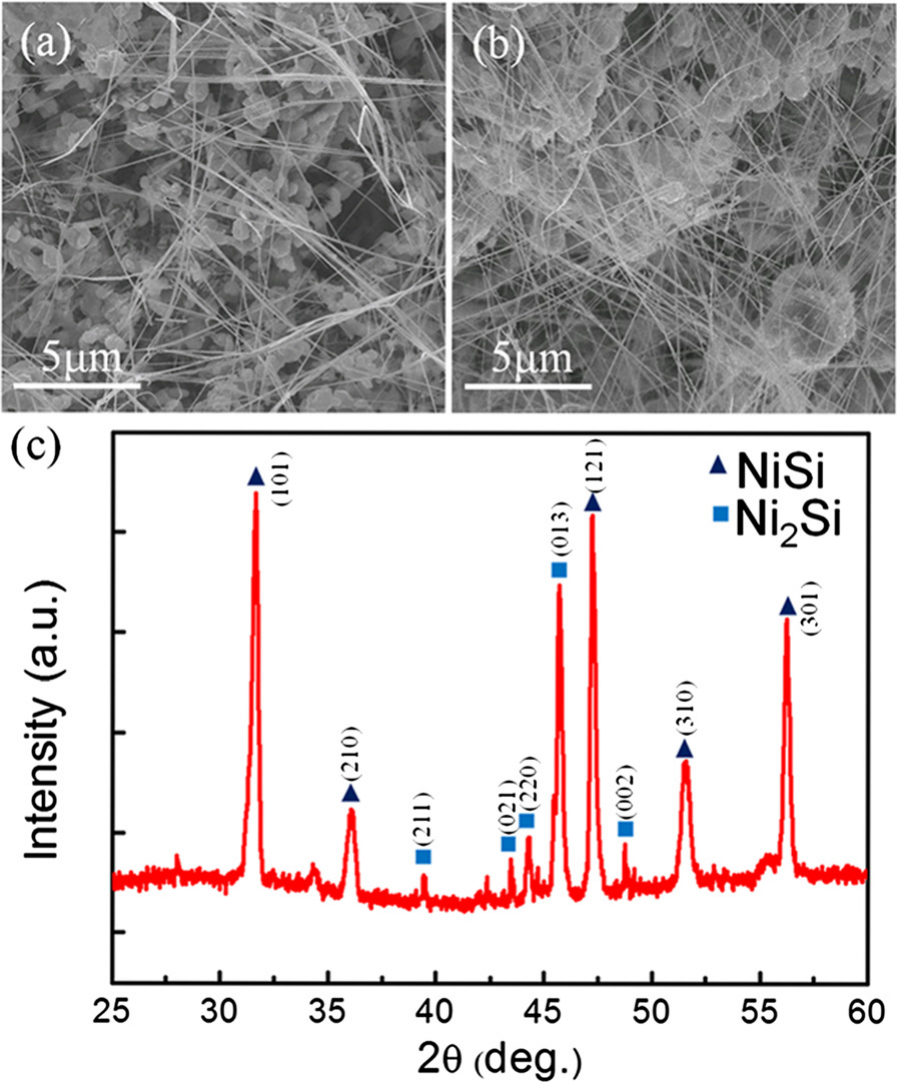
**δ-Ni**_**2**_**Si NWs grown at (a) 15 and (b) 30 min, and (c) corresponding XRD analysis of products.** The temperature was fixed at 400°C, ambient pressure was 9 Torr, and the carrier gas flow rate was 30 sccm.

**Figure 3:**
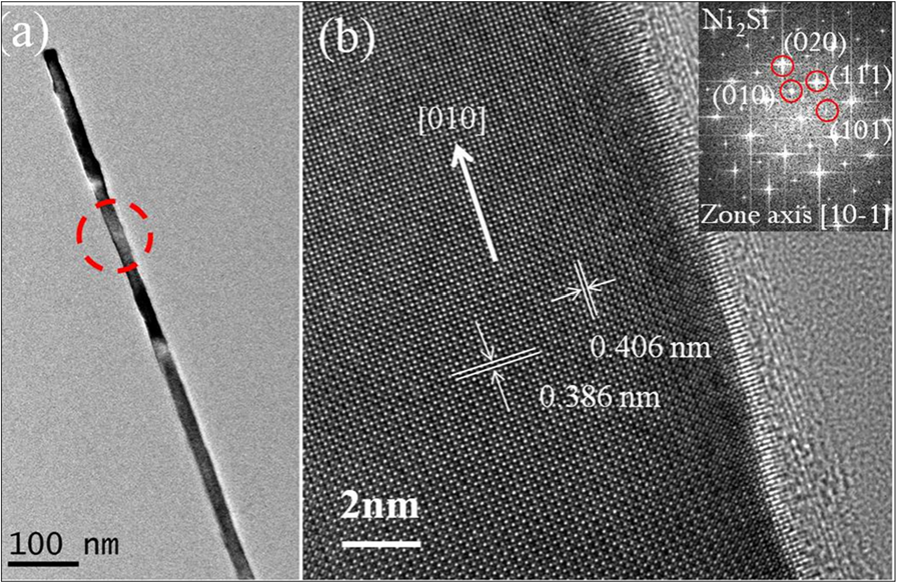
**Low-magnification (a) and high-resolution TEM images (b) of δ-Ni**_**2**_**Si NWs grown at 400°C, 9 Torr, and 30-sccm Ar flow.** The image shows that there exists an oxide layer with 2 nm in thickness on the NW. The inset in (**b**) shows the corresponding FFT diffraction pattern with a [1] zone axis and [010] growth direction.

Figure 2a,b shows a series of SEM images of NWs with different growth times at a constant gas flow rate (30 sccm) and ambient pressure (9 Torr). The yield and density increased prominently when the growth time was raised from 15 to 30 min. The XRD analysis of different reaction time is shown in Figure 2c. The characteristic peaks were examined and identified to be orthorhombic δ-Ni_2_Si and NiSi according to the JCPDF data base. From Figures 1 and 2, SEM images indicate that there were two types of microstructures (NWs and islands) in the products. In order to identify each phase of the microstructures of the as-grown products, structural analysis of the NWs has been performed. Figure 3a is the low-magnification TEM image of the NW with 30 nm in diameter. HRTEM image (Figure 3b) shows the NW of [010] growth direction with 2-nm-thick native oxide. FFT diffraction pattern of the lattice-resolved image is shown in the inset of Figure 3b, which represents the reciprocal lattice planes with [1] zone axis. The phase of the NW has been identified to be δ-Ni_2_Si, constructed with the orthorhombic structure by lattice parameters of *a* = 0.706 nm, *b* = 0.5 nm, and *c* =0.373 nm. Therefore, the as-deposited layer would be ascribed to NiSi.

**Figure 4:**
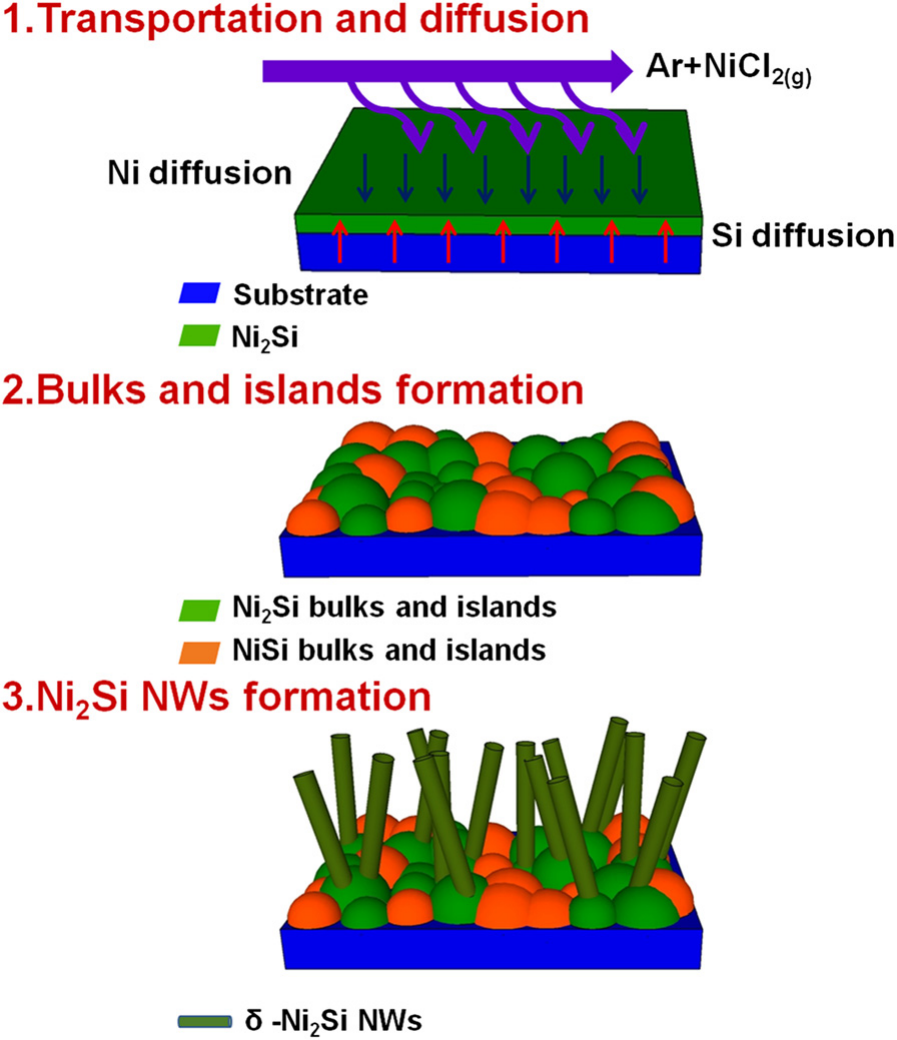
The schematic illustration of the growth mechanism.

The schematic illustration of the growth mechanism is in Figure 4. In the Ni-Si binary alloy system, it has been investigated that Ni atoms are the dominant diffusion species during the growth of orthorhombic δ-Ni_2_Si and NiSi [26]. The reaction and phase transformation between δ-Ni_2_Si and NiSi have also been reported [25]. Based on these previous studies, the reaction of the as-deposited Ni metal film occurred to form δ-Ni_2_Si with a diffusion-controlled kinetics at 300°C to 400°C [27,28]. Then, partial transformation from δ-Ni_2_Si into NiSi thin-film structures could happen if the thickness of the Ni is below 40 nm because NiSi would form on Si substrates with a low Si/NiSi interface energy [26,29]. Then, the continuous supply of Ni atoms may induce further growth of δ-Ni_2_Si phase NWs via surface diffusion kinetics [30] on the remnant δ-Ni_2_Si phase grains or NiSi bulks. There are two plausible and reversible formation paths of δ-Ni_2_Si, which can be described in the following equations [11,24,31]:

(1) $$\mathrm{NiS}i_{\left(s\right)}+\mathrm{NiC}l_{2\left(g\right)}⇄Ni_{2}Si_{\left(s\right)}+Cl_{2\left(g\right)},$$

(2) $$2\mathrm{NiS}i_{\left(s\right)}+2Cl_{2\left(g\right)}⇄Ni_{2}Si_{\left(s\right)}+\mathrm{SiC}l_{4\left(g\right)}.$$

The two equations correspond well with the experiment results: higher ambient pressure will enhance the reaction to form Ni_2_Si according to LeChatelier's principle, contributing to the formation and agglomeration of larger amount of δ-Ni_2_Si NWs and islands at the surface.

**Figure 5:**
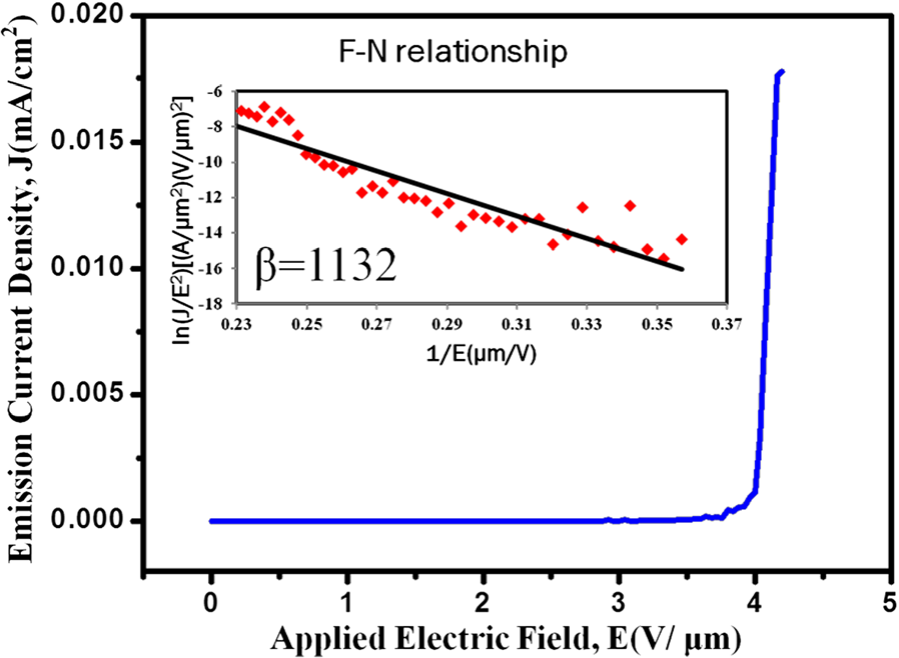
**The field emission plot of δ-Ni**_**2**_**Si NWs.** The inset shows the corresponding ln(*J*/*E*^2^)−1/*E* plot.

Due to the metallic property and special 1-D geometry, investigation of field emission properties has been conducted. Figure 5 shows the plot of the current density (*J*) as a function of the applied field (*E*) and the inset is the ln(*J*/*E*^2^)−1/*E* plot. The sample of δ-Ni_2_Si NWs was measured at 10^−6^ Torr with a separation of 250 μm. According to the Folwer-Nordheim relationship, the field emission behavior can be described by the following equation:

(3) $$J=\left(A\beta^{2}E^{2}/\psi \right)\;\exp\;\left(-B\psi^{3/2}/\mathrm{\beta E}\right).$$

The turn-on field was defined as the applied field attained to a current density of 10 μA/cm^2^ and was found to be 4.12 V/μm for our Ni_2_Si NWs. The field enhancement factor was calculated to be about 1,132 from the slope of the ln(*J*/*E*^2^)−1/*E* plot with the work function of 4.8 eV [32] for Ni_2_Si NWs. Based on the measurements, Ni_2_Si NWs exhibited remarkable potential applications as a field emitter like other silicide NWs [20,25,33].

**Figure 6:**
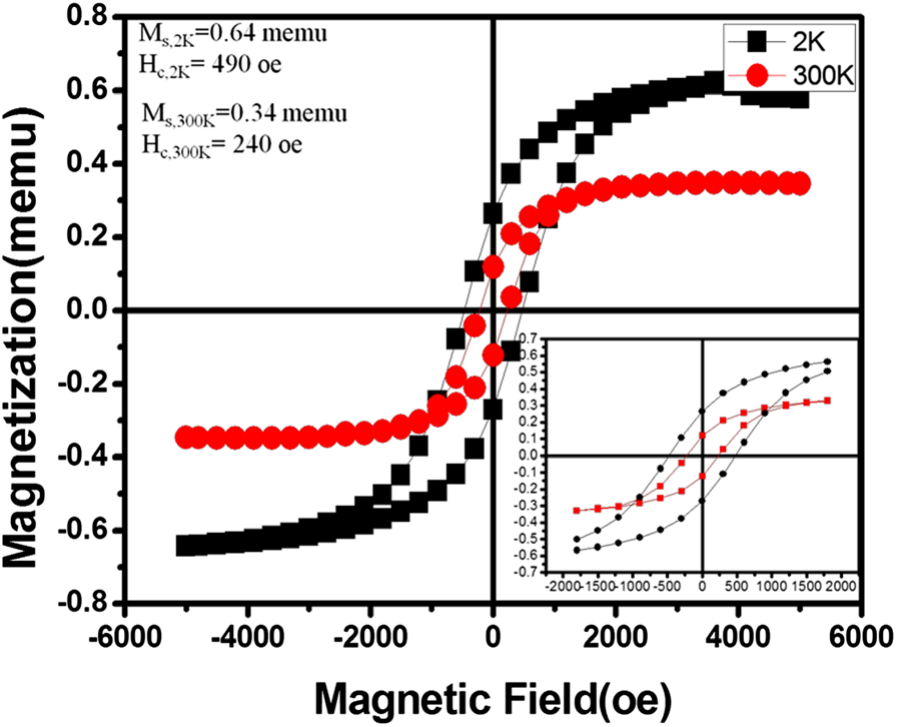
**M-H curve of δ-Ni**_**2**_**Si NWs measured at different temperatures.** The inset is the highlight of the magnetization.

The saturated magnetization (*M*_S_) and coercivity (*H*_C_) of δ-Ni_2_Si NWs were measured using SQUID at 2 and 300 K, respectively. Figure 6 shows the hysteresis loop of the as-grown NWs of 30 nm in diameter with the applied magnetic field perpendicular to the substrates. The inset highlighted the hysteresis loop, which demonstrates a classic ferromagnetic characteristic. The *H*_C_ was measured to be 490 and 240 Oe at 2 and 300 K, respectively, and *M*_S_ was about 0.64 and 0.46 memu, correspondingly. For the magnetization per unit volume (emu/cm^3^), normalization has been introduced through cross-sectional and plane-view SEM images (not shown here) to estimate the density of NWs and the average volume of δ-Ni_2_Si NWs. The estimated values are 2.28 emu/cm^3^ for 2 K and 1.211 emu/cm^3^ for 300 K, respectively. With the normalized value, we may build up a database of the magnetic property of Ni_2_Si NWs, which may be utilized in applications such as cell separation in biology [34].

## Conclusions

δ-Ni_2_Si phase NWs have been successfully synthesized through CVD using a single precursor, NiCl_2_·6H_2_O. The influence of the chamber pressure on the product morphology has been discussed. SEM, TEM, and XRD studies were conducted to analyze the growth mechanism and reaction paths. Electrical measurements show that the field emission property of the δ-Ni_2_Si NWs makes them attractive choices for emitting materials. Magnetic measurements via SQUID at different temperatures show the ferromagnetic property of the δ-Ni_2_Si NWs, and normalization has been applied to calculate the value of magnetization per unit volume. This work has demonstrated future applications of Ni_2_Si NWs on biologic cell separation, field emitters, and magnetic storage.

## Abbreviations

CVD: Chemical vapor deposition; FFT: Fast Fourier transform; HC: Coercivity; HRTEM: High-resolution transmission electronic microscopy; MS: Saturated magnetization; NWs: Nanowires; Oe: Oersted; SQUID: Superconductive quantum interference device; SSPs: Single-source precursors

## Competing interests

The authors declare that they have no competing interests.

## Authors’ contributions

WLC synthesized the Ni_2_Si nanowires. WLC and YTH performed the field emission and magnetization experiments. JYC and CWH analyzed the diffraction data and atomic structure via TEM. CHC analyzed the structure through XRD spectra and demonstrated the illustration of growth mechanism. WLC and WWW conceived the study and designed the research. PHY supported the field emission experiments. WLC, KCL, CLH, and WWW wrote the paper. All authors read and approved the final manuscript.
